# Relationship between spiritual health and hope by dietary adherence in haemodialysis patients in 2018

**DOI:** 10.1002/nop2.412

**Published:** 2019-12-11

**Authors:** Motahareh Musavi Ghahfarokhi, Sara Mohammadian, Bahar Mohammadi Nezhad, Maryam Kiarsi

**Affiliations:** ^1^ Member of Abadan Faculty of Medical Sciences Abadan Faculty of Medical Sciences Abadan Iran; ^2^ Nursing Faculty of Medical Sciences University of Dezful Dezful Iran

**Keywords:** adherence, haemodialysis, hope, nurses, nursing, spiritual well‐being

## Abstract

**Aim:**

The present study aimed to determine the relationship between spiritual well‐being and hope through adherence to diet in haemodialysis patients referred to the dialysis centre.

**Design:**

This research was a cross‐sectional study of a descriptive‐analytical type conducted on 120 patients undergoing haemodialysis in a haemodialysis centre of a hospital affiliated to Dezful University of Medical Sciences using the census method.

**Method:**

The data were collected using a questionnaire and through the laboratory information included in the patient's records. The data collection tools were a demographic information questionnaire, the Ellison and Paloutzian spiritual well‐being scale (SWBS), the Hope‐Herth questionnaire and the objective laboratory criteria (phosphorus, potassium). This included the weight difference between the two dialysis sessions from the record to examine the adherence to the diet.

**Results:**

The results indicate there to be a significant relationship between hope and spiritual well‐being with the objective criteria of the adherence to the diet in dialysis patients (*p* = .001).

## 1 INTRODUCTION

Chronic kidney disease, which is also known as chronic kidney failure, is regarded as one of the most chronic, progressive and life‐threatening diseases in humans, which has become increasingly prevalent the world (Aghighi et al., 2008;Krueger, 2009). This chronic disease is a stage in kidney failure where the nephron function reaches <50% of its normal capacity_._ If the kidney function capacity reaches <10%‐15% of their normal capacity, then they are considered to be in end‐stage renal disease (ESRD). In this stage of the disease, some therapies such as dialysis for the two main types of haemodialysis and peritoneal and kidney transplantation are essential for survival (Gonçalves et al., 2015). Haemodialysis is the most commonly used therapy (more than 90%) in patients with ESRD (Ghods & Savaj, 2006). Currently, there are more than 13,000 dialysis patients in Iran and the rate of these patients increases yearly by 15%, among which 1,500 are dying from the complications of dialysis (Baraz‐Pardenjani, Mohammadi, & Boroumand, 2008; Shafipour, Jafari, Shafipour, & Nasiri, 2010). By increasing the access to dialysis, hundreds of thousands of patients with end‐stage renal failure have a chance to survive, although where this therapy fails to change the course of the disease and it is associated with many physical, psychological and social stresses (Hasanzadeh, Shamsoddini, Moonaghi, & Ebrahimzadeh, 2011). By starting dialysis, different needs and changes may take place in the lives of the patients. Dialysis patients have limitations and adherence requirements concerning certain therapeutic regimens such as fluid and dietary restrictions (low salt, low potassium, low phosphorus, etc.), the proper and real‐time consumption of prescription drugs and regular attendance of the dialysis sessions to maintain their health and to prevent cardiovascular complications (Khalili, Eslami, Farajzadegan, & Hassanzadeh, 2011;Nasiri, 2012). This set of behaviours is described as the adherence to treatment behaviours in dialysis patients (Rambod, Peyrovi, Sareban, & Mohebbi Nubandeghani, 2008). In these patients, the levels of phosphorus and potassium are among the criteria when adopting the diet, due to the effect of diet and interdialytic weight gain, which indicates the recommended amount of salt and fluid consumption. Adherence to the diet and the observation of the restrictions on fluid consumption has led to a reduction in the signs of disease and drug side effects. This plays an influential role in improving their quality of life and it also increases the life expectancy of patients (Durose, Holdsworth, Watson, & Przygrodzkz, 2004)_._

However, the imperfection in adherence to treatments in haemodialysis patients has led to worse illness, increased mortality and costs, in addition to more responsibilities and workload for healthcare providers (Unruh, Evans, Fink, Powe, & Meyer, 2005)_._ The deviance and failure of chronic patients to observe and adhere to dietary restrictions are considered among the major concerns of the healthcare and treatment teams (Hasanzadeh et al., 2011)_._ Kim, Evangelista Lorraine, Phillips Linda, Pavlish, and Kopple Joel (2010) reported the rate of non‐adherence to the diet in haemodialysis patients of approximately 2.1%–4.82%_._ The adherence of patients to the diet is influenced by a variety of factors, including individual attitude and beliefs in treatment, personality and psychological traits, patient information level related to diet, socio‐economic status, cultural differences and the patient's ability to tolerate low consumption of water and fluids (Takaki & Yano, 2006). Since chronic renal failure is a part of the chronic and complex disease, patients experience problems such as daily suffering, dependence on others and dialysis machines and consequently, should undergo continuous diet‐therapy and medication. Therefore, the disease involves a large number of mental instabilities such as anxiety and depression, personality disorder (Theofilou, 2011), sexual function, self‐confidence, beliefs and patient values (Theofilou, 2012b). Further, a spiritual crisis appears in these individuals following the disorder in the patients and in the mechanisms of compliance and communication (Theofilou, 2012a). Some studies have suggested that the spirituality dimension is considered as an essential requirement for these patients (McCord et al., 2004;Puchalski et al., 2011).

Spiritual well‐being is regarded as one of the most important concepts in patients dealing with problems and stresses caused by the disease, which plays a crucial role in arousing the sense of identity, perfection, satisfaction, happiness, beauty, love, respect, positive attitude, inner balance and purpose in life (Marashian & Esmaili, 2012). Spiritual well‐being is known as the latest dimension after the health dimensions such as physical, social and psychological ones, which leads to the integration of other dimensions (Assarroudi, Jalilvand, Oudi, & Akaberi, 2013). This aspect has two existential and religious dimensions. The former is related to the attempt to understand the meaning and purpose of life, while the latter refers to the relationship with a superior power (Dehbashi, Sabzevari, & Tirgari, 2014). Hope is defined as having a positive expectation about the future and a coping strategy for adaptation to dialysis and kidney failure (Allahbakhshian, Jaffarpour, Parvizy, & Haghani, 2010). Further, hope is a cognitive process related to goals and provides the ground for achieving goals (Billington, Simpson, Unwin, Bray, & Giles, 2008). Having hope is a symptom of mental health and its enhancing makes it possible to provide the ground for accepting medical and caring about education from the patient's side (Tutton, Seers, & Langstaff, 2012). Based on the studies conducted by Yi et al. (2006), health and hope are some of the most significant factors in life, which provide the compatibility with the disease, reduce mental health and improve quality of life and psychosocial well‐being. Schiavon, Marchetti, Gurgel, Busnello, and Reppold (2016) assumed individuals with greater optimism and hope seek to engage in healthier behaviours, which greatly contributes to chronic disease treatment, regardless of their clinical status. On the other hand, despair and hopelessness and non‐adherence to the treatment play an important role in chronic diseases and are still a challenge in the medical and social professions (Dulmen et al., 2007).

### 1.1 Aim

The non‐adherence to the diet in dialysis patients has led researchers to explore relevant factors affecting its adherence. Identifying the factors related to the non‐adherence to the diet is the first step in determining patients at risk of non‐adherence and recognizing related factors (Lurie et al., 2000).

The present study seeks to identify the relationship between spiritual well‐being and hope with diet‐adherence in haemodialysis patients referred to the dialysis centre, in Dezful County. Furthermore, this research evaluates diet‐adherence and the factors affecting it. This plays an influential role in promoting health, preventing deterioration and reducing mortality of patients.

## 2 METHOD

### 2.1 Sampling procedure

The statistical population included the patients undergoing haemodialysis in the haemodialysis centre of the hospital affiliated to the Dezful University of Medical Sciences. The sampling process was performed by the census method according to the previous studies and the number of dialysis patients in the centre. The study population involved 140 patients treated with dialysis at the haemodialysis centre; 15 patients were excluded due to being of a very high or low age or having a lack of ability to communicate their responses to the questionnaires. Furthermore, five patients expressed reluctance and did not participate in the study. Finally, 120 patients who met the criteria for entering the study were selected. The inclusion criteria were those who consented to participate in the study, who had at least 3 months of haemodialysis history, who were aged over 18, who have 3–4 hr of dialysis per session at least twice weekly. They also had to have the ability to understand questions or to read and write. The exclusion criteria included any mental or cognitive disorder and dissatisfaction concerning participating in the study. If the patient was not able to fill out the questionnaire because of a lack of literacy or the inability to write, then the questions were read and explained to the patient by the researchers' colleagues.

### 2.2 Data collection procedure

The present study is an analytical‐descriptive and cross‐sectional study. The data were collected during December 2017 and February 2018, using information questionnaires (age, sex, education, income level, occupation, duration of haemodialysis and underlying disease), Ellison and Paloutzian spiritual well‐being scale (SWBS) and Hope‐Herth questionnaire. In addition, the objective laboratory criteria (phosphorus and potassium) and weight difference between the two dialysis sessions from the patients’ records were used for examining the adherence to the diet.

### 2.3 Measurement of variables

The spiritual well‐being questionnaire was designed by Palutzian and Ellison (1982) and included 20 questions. Questions with even and odd numbers assessed the existential and religious well‐being, respectively and ultimately, the total score of the spiritual well‐being was calculated from the total sum. The answer to the questions was a six‐point Likert scale from “Strongly disagree” to “Strongly agree.” In questions with positive nature, the numbers 1 and 6 were given to “Strongly disagree” and “Strongly agree,” while this process was reversed in questions with negative nature such that numbers 1 and 6 were given to “Strongly agree” and “Strongly disagree,” respectively. Therefore, the range of spiritual well‐being scores was between 20 and 120 and the existential and religious spirituality were investigated between 10 and 60, respectively. The validity and reliability of the questionnaire were evaluated and validated by Palutzian and Ellison (1982) and Cronbach's alpha coefficient was calculated at 0.91, 0.91 and 0.93 for the religious and existential well‐being and total scales, respectively. Regarding the conducted studies (e.g. Assarroudi et al., 2013; Dehbashi et al., 2014; Moghimiyan & Salmani, 2010), spiritual well‐being scores were classified into three levels of low (20–40), moderate (41–99) and high (120–100). Baljani, Khoshabi, Amanpour, and Azimi (2011) reported that Cronbach's alpha indicated acceptable reliability (*r* = .93), which was confirmed by Rezaie, Seyyed Fatemi, and Hosseini (2008) (*r* = .79).

The Herth‐Hope questionnaire was an American easy‐to‐understand self‐report tool, which was written by Herth in 1992 and included 12 phrases with different degrees (Agree, Disagree and Unsure) and scores (1–3). In this questionnaire, the order of scoring questions 3 and 6 was such that the scores 1–3 were given to Agree, Unsure and Disagree, respectively. However, other questions were scored from 3 to 1, respectively. Scores 12–24 were considered as low hope, 25–30 as average hope and 31–36 as high hope. In general, the minimum and maximum scores of 12 and 36 were considered for each person. The Cronbach's alpha coefficient and the reliability of this questionnaire were calculated at 0.97 and 0.91 by Herth (1992) with a test–retest method for 2 weeks. Baljani et al. (2011) confirmed the validity of the questionnaire and the reliability of the questionnaire was calculated at 0.82 with Cronbach's alpha.

The values of laboratory indexes (potassium, phosphorus and interdialytic weight gain) were employed to study the adherence to the diet, which was defined as K < 6, PO4 < 7 mg/dl and the interdialytic weight gain (*IDWG*)^9^ < 2 (43) in three short dialysis interval. Moreover, interdialytic weight gain (IDWG) is an objective index for the adherence to the restrictions on fluids consumption. Height and weight measurements were computed using calibrated electronic scales and a stadiometer and the body mass index (BMI) was calculated by dividing weight (kg) by the height (m^2^). IDWG is the result of a difference in patient's weight immediately after the end of the dialysis session and before the start of the next dialysis session. The average of this index during a recent month was recorded in the patient's record. Based on the previous studies, the average amount of phosphorus and potassium during the past 3 months was calculated as the laboratory indexes of adherence to the restrictions on potassium and phosphorus (Esmaeili, Ahmadi, Jannati, Khalilian, & Espabodi, 2013;Rambod et al., 2008). If their mean values were higher than the normal one, they were considered as non‐adherent to the treatment. According to Kugler, Vlaminck, Haverich, and Maes (2005) and Esmaeili et al. (2013), serum potassium levels >6 mg/dl indicated the lack of adherence to the low‐potassium regime while serum phosphorus levels >7 mg/dl represented the non‐adherence to the low‐phosphorus regimen. The interdialytic weight gain more than 2 kg relative to the patient's dry weight was a sign of non‐adherence to the restrictions on fluid consumption. Accordingly, patients were categorized into adherent and non‐adherent to the low‐potassium, low‐phosphorus regimes and restrictions on fluid consumption (Kugler et al., 2005).

### 2.4 Data analysis

Descriptive statistics were employed to evaluate the mean, standard deviation, frequency and range of changes in demographic variables, hope, spiritual well‐being and its dimensions, as well as objective criteria of the adherence to the diet. To determine the relationship between variables, the chi‐square test and Pearson correlation coefficient were used for qualitative and quantitative data, respectively. The data were analysed using SPSS software (version 16).

### 2.5 Ethical issues

The ethics code and hospital entrance permission were received from the University Ethics Committee. In this study, researchers were committed to ethical issues of obtaining informed written consent from participants, respect for voluntary participation and informing the participants of the purpose of the study. Also, the patients were assured that questionnaire information was completed in an anonymous and confidential way.

## 3 RESULTS

The number of patients that participated in this study totalled 110 with an average age of 53.90 (*SD* 13.65) years, most of whom were over the age of 59 (40%). Furthermore, most patients were women (57 patients, 51.8%) and married 84.5% (93 people). In terms of education, 50% (55 patients) were illiterate or had an elementary level of education. Additionally, 59.1% (65 patients) of patients were unemployed and 52.7% (58 patients) reported that their incomes were about 5,000 thousand Rials a month. In addition, 48.2% (53 patients) of patients had a haemodialysis history between 1–5 years and 50.9% had high blood pressure (Table 1).

**Table 1:** Distribution of absolute and relative abundance of the research units based on the personal characteristics

Demographic characteristics	Groups	Frequency	Percentage
Age	Under 30	6	5.5
	30–39	11	10.0
	40–49	16	14.5
	50–59	33	30.0
	Above 59	44	40.0
Gender	Male	53	48.2
	Female	57	51.8
Marital status	Single	17	15.5
	Married	93	84.5
Education level	Illiterate and elementary	55	50.0
	Middle school and high school	30	27.3
	Diploma	19	17.3
	Above diploma	6	5.5
Income level	5,000 Thousand Rials	58	52.7
	10 Million – 5,000 Thousand Rials	24	21.8
	Above 1 Million Rials	28	25.5
Job	Unemployed	65	59.1
	Worker	18	16.4
	Employee	13	11.8
	Self‐employment	14	12.7
History of haemodialysis	1 year	36	32.7
	1–5 years	53	48.2
	Above 5 years	21	19.1
Underlying disease	High blood pressure	56	50.9
	Diabetes	20	18.2
	Diabetes and blood pressure	34	30.9

The results indicated that the mean score for hope was 30.10 (*SD* 5.82) among the dialysis patients. Most patients (69 patients, 62.7%) had a high level of hope while 17.3% (19 patients) had moderate hope and 20% (22 patients) had low hope. In terms of spiritual well‐being, the mean score of participants was obtained 75.05 (*SD* 7.54). Furthermore, 78.2% of patients (86 patients) had a moderate score, 19.1% (21 patients) had a high score and 2.7% (3 patients) had a low score in terms of their spiritual well‐being. Regarding the objective method of assessing the adherence of dialysis patients to their diet, the data indicated that 69.1% (76 patients) had an interdialytic weight gain of <2 kg during the last month. Generally, 89.1% (98 patients) adhered to the low‐potassium diet (potassium <6 mEq/l over the last 3 months) and 65.5% (72) adhered to the low‐phosphorus diet (phosphorus <7 mEq/l over the last 3 months) (Table 2).

**Table 2:** Distribution of absolute and relative frequency, mean and standard deviation, and range of life expectancy, spiritual well‐being and adherence of the diet in research units

Variable		Categories	Frequency	Percentage	Mean and standard deviation	Variation range
Hope		Low	22	20.0	30.10 ± 5.82	14–36
		Moderate	19	17.3		
		High	69	62.7		
Spiritual well‐being		Low	3	2.7	75.05 ± 7.54	58–108
		Moderate	86	78.2		
		High	21	19.1		
Adherence to the diet	Interdialytic weight gain	Adherence	76	69.1	1.85 ± 1.06	0.20–6
		Non‐adherence	34	30.9		
	Serum potassium level	Adherence	98	89.1	5.13 ± 0.75	2.90–6.60
		Non‐adherence	12	10.9		
	Serum phosphorous level	Adherence	72	65.5	5.46 ± 1.38	2.80–9.5
		Non‐adherence	38	34.5		

In the process of determining the relationship between hope and spiritual well‐being with dietary adherence using the objective method, the results showed a significant relationship between hope and interdialytic weight gain (*r* = −.289, *p* = .002), the number of absences in terms of the haemodialysis sessions (*r* = −.443, *p* = .001), serum potassium level (*r* = −.293, *p* = .012) and serum phosphorus level (*r* = −.328 and *p* = .001). Based on the results of the Pearson correlation between spiritual well‐being and adherence to diet, a significant relationship was observed between spiritual well‐being and interdialytic weight gain (*r* = −.328, *p* = .001), serum potassium level (*r* = −.365, *p* = .001) and serum phosphorus level (*r* = −.430 and *p* = .001). Furthermore, a significant relationship was identified between the dimensions of spiritual well‐being and adherence to the diet, as well as between religious and existential well‐being with the index of interdialytic weight gain, in addition to the number of absences in haemodialysis sessions, serum potassium and serum phosphorus levels (*p* < .05) (Table 3).

**Table 3:** The relationship between life expectancy, spiritual well‐being and dietary adherence in research units

Variable	Interdialytic weight gain		Serum potassium level		Serum phosphorus level	
	*r*	*p*	*r*	*p*	*r*	*p*
Hope	.289**	.002	.239*	.012	.328**	.001
Spiritual well‐being	.307**	.001	.365**	.001	.430**	.001
Religious well‐being	.291**	.002	.173	.07	.266**	.005
Existential well‐being	.215*	.024	.420**	.001	.436**	.001

** Significant at *p* < .01.

* Significant at .05.

As shown in Tables 4 and 5, no significant relationship was found between hope, spiritual well‐being, the existential and religious dimensions of spiritual well‐being and the objective indicators of the adherence to the diet with variables such as age, education, income and occupation and the history of haemodialysis at a level of 0.05. However, there was a significant relationship between spiritual as well as existential well‐being and the underlying disease (*r* = −.204 and *p* = .032, *r* = −.188 and *p* = .049).

**Table 4:** The relationship between hope, spiritual well‐being and adherence to the diet with demographic characteristics in research units

Variable	Age		Education		Income level		Job	
	*r*	*p*	*r*	*p*	*r*	*p*	*r*	*p*
Hope	−.009	.92	.055	.56	.157	.10	−.134	.16
Spiritual well‐being	.001	.99	.128	.18	.083	.39	.047	.62
Religious well‐being	.043	.65	.091	.34	−.041	.66	.109	.25
Existential well‐being	−.041	.67	.119	.21	.093	.33	−.028	.77
Interdialytic weight gain (kg)	−.101	.29	−.055	.56	−.096	.31	.066	.49
Serum potassium level	−.016	.87	−.113	.24	.021	.82	−.014	.88
Serum phosphorus level	−.036	.71	.038	.69	−.002	.98	.026	.78

**Table 5:** The relationship between life expectancy, spiritual well‐being and adherence to the diet with demographic characteristics in research units

Variable	Gender		Marital status		Haemodialysis duration		Underlying disease	
	*χ* ^2^	*p*	*χ* ^2^	*p*	*r*	*p*	*r*	*p*
Hope	0.038	.98	4.62	.099	.094	.32	.048	.61
Spiritual well‐being	2.95	.22	3.78	.15	.092	.33	−.204*	.032
Religious well‐being	1.110	.57	1.93	.38	.062	.52	−.146	.12
Existential well‐being	0.247	.88	4.49	.10	.089	.35	−.188*	.049
Interdialytic weight gain (kg)	1.95	.16	1.65	.19	.078	.41	.108	.25
Serum potassium level	0.229	.63	2.46	.11	.014	.88	.011	.91
Serum phosphorus level	0.015	.90	1.07	.29	.122	.20	.065	.50

* Significant at *p* < .05.

## 4 DISCUSSION

Considering the importance of adherence to dietary restrictions in haemodialysis patients and the risk of adverse complications caused by non‐compliance with these limitations, the present study was conducted to evaluate the relationship between spiritual well‐being and hope with adherence to the diet in haemodialysis patients, with the aim of determining the role of the involved factors in enhancing the adherence to the diet.

In terms of dietary adherence based on the objective indexes, including potassium, phosphorus and being overweight between the two sessions, most patients were among the adherence to the diet group. The present study represented that most patients (89.1%) had a normal range of potassium, indicating that most patients were adherent to the low‐potassium diet. In this regard, this research is similar to the studies conducted by Gibson, Held, Khawnekar, and Rutherford (2016) and Esmaeili et al. (2013), which showed a potassium level in the range of 60%–96% in dialysis patients. It is worth noting that 65.5% of patients were in the expected range for their phosphorus level and they were adherent to the low‐phosphorus diet. This is consistent with the results of some of the studies that reported the phosphorus range of the patients as being between 43% and 81%. However, Lee and Molassiotis (2002) emphasized the rate of the patients’ adherence to a low‐phosphorus diet by 43%, which is not consistent with the results of this paper. The lower adherence of the patients to the low‐phosphorus diet suggests that the patients have no adherence to the medical diet in addition to the non‐observance of restrictions in terms of phosphorus. Compared with the low‐phosphorus diet in the present study, the higher adherence of patients to a low‐potassium diet may indicate that the patients are aware of the dangerous complications of potassium such as cardiac arrest and death, although the complications of phosphorus are revealed gradually and they are not as dangerous as potassium. The results of this research indicate the adherence of most patients (69.1%) to the fluid regimes and the preservation of an interdialytic weight gain of <2 kg. This is in line with the studies of Esmaeili et al. (2013) and Gibson et al. (2016). In this regard, the results of some of the other studies (Lee & Molassiotis, 2002; Rambod, Peyrovi, Sareban & Mohebbi., 2008) that reported on the rate of non‐adherence to the fluid regime by 59.7% and 56%, respectively, were different from the results of the present study. Factors such as the amount of urine left, the dialysis hours and the interval between two sessions of dialysis affects the interdialytic weight gain (Esmaeili et al., 2013). In addition, the cutting point in the classification of the groups into two categories (adherence and non‐adherence) is different across the various studies (Khalili et al., 2011;Rambod et al., 2008).

The results highlighted that most patients had a high level of hope, which is consistent with the studies of Ottaviani et al. (2014) and which measured the hope of patients undergoing haemodialysis. Considering the study conducted by Alberto and Joyner (2008) on chronic obstructive pulmonary disease, the hope score was reported to be at a high level between 35 and 48, although this score was reported at a low level in the studies of Baljani et al. (2011) and Dehbashi et al. (2014). The difference in the results of these two studies with those in the present study can be attributed to the cultural differences affecting the acceptance of the disease, the quality of medical care and the level of perceived social support. In addition to these factors, since most the patients had a history of 1–5 years of haemodialysis treatment, it is possible to highlight the effectiveness of using the mechanisms of adaptation to chronic disease and passing the disease crisis in relation to the high level of hope in patients undergoing haemodialysis.

Based on the results, the scores of spiritual well‐being and its existential and religious dimensions were moderate in most patients. This is consistent with the results in the studies of Baljani et al. (2011) and Dehbashi et al. (2014). However, the spiritual well‐being scores were at a high level in other studies (Hojjati, Qorbani, Nazari, Sharifnia, & Akhundzadeh, 2010;Taheri kharameh et al., 2013). Considering the similar conditions of age in these studies with the present study, the difference in the results can be attributed to the differences in the research environments and the ethnic groups of the participants. This is because different geographic environments do not result in the same spiritual well‐being due to differences in the religious backgrounds.

By studying the relationship between spiritual well‐being and adherence to diet, the results demonstrated a significant statistical relationship between the two dimensions of spiritual well‐being including existential and religious well‐being and the objective indexes of the adherence to the diet (potassium, phosphorus, interdialytic weight gain, as well as the number of absences in dialysis sessions). Based on the results, spiritual well‐being is an important factor in the context of the adherence to diet in dialysis patients. Individuals with a higher spiritual well‐being were more adherent to their diet, although no significant relationship was observed between religion and adherence to the treatment in the study of Berman et al. (2004). This lack of relationship, according to the researcher, could be attributed to a small number (about 5) of patients who denied belonging to any religion (Berman et al., 2004). Alvarez et al. (2016) found there to be a statistically significant relationship between spirituality and adherence to treatment in patients with heart failure. Black, Davis, Heathcotte, Mitchell, & Sanderson, (2006) suggested that spiritual beliefs affect health beliefs, which could lead to health activities such as using the prescribed drugs, weight control and adherence to and compliance with the therapeutic regime. The findings of the present study highlight the significant relationship between hope and all of the objective indexes of the adherence to the diet (potassium, phosphorus and interdialytic weight gain). Hope is regarded as one of the most important factors in the effective adaptation to chronic disease, as well as the acceptance of the therapeutic regimen (Eliott & Olver, 2009). Hope is a strong adaptation mechanism in chronic patients and hopeful people can easily cope with the crisis of disease. Furthermore, hope is the centre of desire and attention in individuals and it is an essential factor for survival and continuity of life (Ghezelseflo & Esbati, 2013). Berg, Rapoff, Snyder, and Belmont (2007) demonstrated that the existence of psychological variables, especially hope, could lead to an increase in the adherence to treatment in children with asthma.

The results of the present study indicate there to be a significant statistical relationship between hope and spiritual well‐being. Accordingly, the higher the level of spiritual well‐being in haemodialysis patients, the higher the level of hope will be. In addition, Ottaviani et al. (2014) and Dehbashi et al. (2014) showed there to be a significant relationship between spiritual well‐being and hope in haemodialysis patients. In another study, Pinto (2012) found a positive and moderate correlation between hope and spiritual well‐being among cancer patients. Thus, hope is regarded as one of the essential requirements of human beings, that creates the power of support, encouragement and adaptation to hard conditions in one person. However, religion and spirituality may be associated with hope. Since the dimensions of spirituality make life meaningful and they are considered to be a source of hope, spirituality leads to adaptation to disease (Aghighi et al., 2008). By reviewing the studies conducted in the domain of hope, Souliotis et al. (2016) reported that high levels of hope have a positive relationship with physical and psychological well‐being, high self‐esteem, positive thinking and extraordinary social relationships.

It should be noted that no significant relationship was found between the objective indicators of the adherence to the diet and demographic variables. This is like the results of Esmaeil et al. (2013) regarding the non‐existence of a significant relationship between the amount of potassium and phosphorus and the demographic characteristics of the patients. This difference can be related to the low level of education of most people (illiterate and elementary) in the present study. However, Rambod, Peyrovi, Sareban and Mohebbi (2008) concluded there to be a significant relationship between education level and the amount of phosphorus, potassium, urea and interdialytic weight gain.

Regarding the examination of the relationship between the hope and demographic variables, no significant relationship was found between hope and sex, marital status, the duration of haemodialysis and underlying disease. Furthermore, no significant relationship was observed between spiritual well‐being and the variables such as gender, marital status and the duration of haemodialysis. Subsequently, there was a significant statistical difference between spiritual well‐being and underlying disease. Based on this interpretation, people with a higher spiritual well‐being have better compatibility and control over underlying diseases such as blood pressure and diabetes. People may be encouraged to bolster their spirituality in order to find the additional motivation to begin and maintain healthy behaviours (Aldwin, Park, Jeong, & Nath, 2014).

## 5 LIMITATIONS OF THE STUDY

The limitations of the present study included the limited sample size and addressing only one centre, which restricted the generalizability of the findings. On the other hand, some limitations refer to the cross‐sectional study design. This is as it does not allow for us to determine a causal relationship between spiritual health and hope with dietary adherence. Furthermore, hope and spirituality health, as well as diet and fluid adherence, can be changed in different conditions over time. The longitudinal follow‐up of these patients is in progress and it will hopefully allow us to be better able to delineate this relationship. Finally, the objective index was only used to assess the dietary and fluid adherence and no information was available on the subjective aspects of adherence in these patients. In general, access to this type of information could be useful to determine the behaviours related to adherence to diet and fluid limitations better. Since this research was a simple analytical‐descriptive study conducted over a short period of time using the census method with a single group, we had some limitations when it came to controlling all the confounders. For more precision and control of the potential confounders, conducting interventional studies using random sampling in a larger population is recommended.

## 6 CONCLUSION

The present study showed that hope and spirituality could be essential variables and are related to diet‐adherence. These issues warrant further investigation. It is suggested that future studies could examine a larger community of chronic dialysis patients as well as other chronic diseases. It could also consider all aspects of the adherence to the therapeutic regimen, both subjectively and objectively. Finally, approaches should be considered to increase the level of hope and spiritual well‐being in these patients.

## CONFLICT OF INTEREST

No conflict of interest has been declared by the authors.

## References

[nop2412-bib-0001] Aghighi, M. , Heidary Rouchi, A. , Zamyadi, M. , Mahdavi‐Mazdeh, M. , Rajolani, H. , Ahrabi, S. , & Zamani, M. (2008). Dialysis in Iran. Iranian Journal of Kidney Diseases, 2(1), 5–11. 19367003

[nop2412-bib-0002] Alberto, J. , & Joyner, B. (2008). Hope, Optimism and self‐care among better breathers Support group members with chronic obstructive pulmonary disease. Applied Nursing Research, 21(4), 207–212. 10.1016/j.apnr.2006.12.005 18995163

[nop2412-bib-0003] Aldwin, C. M. , Park, C. L. , Jeong, Y. J. , & Nath, R. (2014). Differing pathways between religiousness, spirituality and health: A self‐regulation perspective. Psychology of Religion and Spirituality, 6(1), 9 10.1037/a0034416

[nop2412-bib-0005] Allahbakhshian, M. , Jaffarpour, M. , Parvizy, S. , & Haghani, H. (2010). A Survey on the relationship between spiritual wellbeing and quality of life in multiple sclerosis patients. Zahedan Journal of Research in Medical Sciences, 12(3), 29–33.

[nop2412-bib-0006] Alvarez, J. S. , Goldraich, L. A. , Nunes, A. H. , Zandavalli, M. C. B. , Zandavalli, R. B. , Belli, K. C. , … Clausell, N. (2016). Association between spirituality and adherence to management in outpatients with heart failure. Arquivos Brasileiros De Cardiology, 106(6), 491–501. 10.5935/abc.20160076 PMC4940148 27192385

[nop2412-bib-0007] Assarroudi, A. , Jalilvand, M. R. , Oudi, D. , & Akaberi, A. (2013). The relationship between spiritual well‐being and life satisfaction in the nursing staff of Mashhad Hasheminezhad Hospital. Modern Care Journal, 9(2), 156–162.

[nop2412-bib-0008] Baljani, E. , Khoshabi, J. , Amanpour, E. , & Azimi, N. (2011). A study of the relation between spiritual health, religion and hope in cancer patients. Quarterly Journal of Hayat, 17(3), 27–37.

[nop2412-bib-0009] Baraz‐Pardenjani, S. , Mohammadi, E. , & Boroumand, B. (2008). The effect of self–care teaching by videotape on physical problems and quality of life in dialysis patients. Iran Journal of Nursing, 21(54), 121–133.

[nop2412-bib-0010] Berg, C. J. , Rapoff, M. A. , Snyder, C. R. , & Belmont, J. M. (2007). The relationship between children's hope for pediatric asthma treatment adherence. The Journal of Positive Psychology, 2(3), 176–184.

[nop2412-bib-0011] Berman, E. , Merz, J. F. , Rudnick, M. , Snyder, R. W. , Rogers, K. K. , Lee, J. , … Lipschutz, J. H. (2004). Religiosity in a hemodialysis population and its relationship to satisfaction with medical care, satisfaction with life and adherence. American Journal of Kidney Diseases, 1(44), 3 10.1016/S0272-6386(04)00818-2 15332222

[nop2412-bib-0012] Billington, E. , Simpson, J. , Unwin, J. , Bray, D. , & Giles, D. (2008). Does hope predict adjustment to end‐stage renal failure and consequent dialysis? British Journal of Health Psychology, 13, 683–699. 10.1348/135910707X248959 17958929

[nop2412-bib-0013] Black, G. , Davis, B. A. , Heathcotte, K. , Mitchell, N. , & Sanderson, C. (2006). The relationship between spirituality and compliance in patients with heart failure. Progress in Cardiovascular Nursing, 21(3), 128–133. 10.1111/j.0889-7204.2006.04804.x 16957458

[nop2412-bib-0015] Dehbashi, A. , Sabzevari, S. , & Tirgari, B. (2014). The relationship between spiritual well‐being and hope in Hemodialysis patients referring to the Khatam Anbiya hospital in Zahedan 2013‐2014. Medical Ethics, 8(30), 77–97.

[nop2412-bib-0016] Dulmen, D. , Sluijs, E. , Dijk, L. , Ridder, D. , Heerdink, R. , & Bensing, J. (2007). Patient adherence to medical treatment. BMC Health Services Research, 7, 55. 17439645 10.1186/1472-6963-7-55 PMC1955829

[nop2412-bib-0017] Durose, O. C. , Holdsworth, M. , Watson, V. , & Przygrodzkz, F. (2004). Knowledge of dietary restrictions and the medical consequences of non‐compliance by patients on hemodialysis are not predictive of dietary compliance. Journal of the American Dietetic Association, 104, 35–41. 10.1016/j.jada.2003.10.016 14702581

[nop2412-bib-0018] Eliott, J. A. , & Olver, I. N. (2009). Hope, life and death: A qualitative analysis of dying cancer patients' talk about hope. Death Studies, 33(7), 609–638. 10.1080/07481180903011982 19623763

[nop2412-bib-0019] Esmaeili, R. , Ahmadi, H. , Jannati, Y. , Khalilian, A. R. , & Espabodi, F. (2013). The relationship between perceived social support and self‐efficacy with diet adherence among hemodialysis patient. Scientific Journal of Hamadan Nursing & Midwifery Faculty, 21(3), 59–67.

[nop2412-bib-0020] Ghezelseflo, M. , & Esbati, M. (2013). Effectiveness of hope‐oriented group therapy on improving quality of life in HIV male patients. Procedia‐ Social and Behavioral Sciences, 84, 534–537. 10.1016/j.sbspro.2013.06.599

[nop2412-bib-0021] Ghods, A. , & Savaj, S. (2006). Iranian model of paid regulated living‐unrelated kidney donation. Clinical Journal of the American Society of Nephrology, 1(6), 1136–1145. 17699338 10.2215/CJN.00700206

[nop2412-bib-0022] Gibson, E. L. , Held, I. , Khawnekar, D. , & Rutherford, P. (2016). Differences in knowledge, stress, sensation seeking and locus of control linked to dietary adherence in hemodialysis patients. Frontiers in Psychology, 7, 1–10. 10.3389/fpsyg.2016.01864 27965605 PMC5126042

[nop2412-bib-0023] Gonçalves, F. A. , Dalosso, I. F. , Borba, J. M. C. , Bucaneve, J. , Valerio, N. M. P. , Okamoto, C. T. , & Bucharles, S. G. E. (2015). Quality of life in chronic renal patients on hemodialysis or peritoneal dialysis: A comparative study in a referral service of Curitiba‐PR. Brazilian Journal of Nephrology, 37(4), 467–474. 10.5935/0101-2800.20150074 26648496

[nop2412-bib-0024] Hasanzadeh, F. , Shamsoddini, S. , Moonaghi, H. K. , & Ebrahimzadeh, S. (2011). A comparison of face to face and video‐based education on attitude related to diet and fluids adherence in hemodialysis patients. Ofogh‐e‐Danesh; Journal of Gonabad University of Medical Sciences, 11(4), 34–43.

[nop2412-bib-0025] Herth, K. (1992). Abbreviated instrument to measure hope: Development and psychometric evaluation. Journal of Advanced Nursing, 17(10), 1251–1259. 10.1111/j.1365-2648.1992.tb01843.x 1430629

[nop2412-bib-0026] Hojjati, H. , Qorbani, M. , Nazari, R. , Sharifnia, S. H. , & Akhundzadeh, G. (2010). On the relationship between prayer frequency and spiritual health in patients under hemodialysis therapy. Journal of Fundamentals of Mental Health, 12(2), 514–521.

[nop2412-bib-0027] Khalili, F. , Eslami, A. A. , Farajzadegan, Z. , & Hassanzadeh, A. (2011). The association between social‐ psychological factors and treatment adherence behaviors among maintenance hemodialysis patients in Isfahan, Iran: A conceptual framework based on social cognitive theory. Journal of Health System Research, 7(3), 278–290.

[nop2412-bib-0028] Kim, Y. , Evangelista Lorraine, S. , Phillips Linda, R. , Pavlish, C. , & Kopple Joel, D. (2010). The end‐stage renal disease adherence questionnaire (ESRDAQ): Testing the psychometric properties in patients receivingin‐center hemodialysis. Nephrology Nursing Journal, 37(4), 377–393. 20830945 PMC3077091

[nop2412-bib-0029] Krueger, L. (2009). Experiences of Hmong patients on hemodialysis and the nurses working with them. Nephrology Nursing Journal, 36(4), 379–387. 19715106

[nop2412-bib-0030] Kugler, C. , Vlaminck, H. , Haverich, A. , & Maes, B. (2005). Nonadherence with diet and fluid restrictions among adults having hemodialysis. Journal of Nursing Scholarship, 37(1), 9–25. 10.1111/j.1547-5069.2005.00009.x 15813583

[nop2412-bib-0031] Lee, S. H. , & Molassiotis, A. (2002). Dietary and fluid compliance in Chinese hemodialysis patients. International Journal of Nursing Studies, 39(7), 695–704. 10.1016/S0020-7489(02)00007-X 12231026

[nop2412-bib-0032] Lurie, S. , Shemesh, E. , Sheiner, P. A. , Emre, S. , Tindle, H. L. , Melchionna, L. , & Shneider, B. L. (2000). Non‐adherence in pediatric liver transplant recipients – An assessment of risk factors and natural history. Pediatric Transplantation, 2, 200–201. 10.1034/j.1399-3046.2000.00110.x 10933320

[nop2412-bib-0033] Marashian, F. , & Esmaili, E. (2012). Relationship between religious beliefs of students with mental health disorders among the students of the Islamic Azad University of Ahvaz. Procedia ‐ Social and Behavioral Sciences, 46, 1831–1833. 10.1016/j.sbspro.2012.05.387

[nop2412-bib-0034] McCord, G. , Gilchrist, V. J. , Grossman, S. D. , King, B. D. , McCormick, K. E. , & Oprandi, A. M. , … Srivastava, M. (2004 ). Discussing spirituality with patients. A rational and ethical approach. Annals of Family Medicine, 2(4), 356–361. 15335136 10.1370/afm.71 PMC1466687

[nop2412-bib-0035] Moghimiyan, M. , & Salmani, F. (2010). Correlation between spiritual wellbeing and hope in cancer patients referred to Isfahan University of medical sciences. Qom University of Medical Sciences Journal, 6(3), 40–45.

[nop2412-bib-0036] Nasiri, A. (2012). Effect of an educational plan based on the Teach‐Back method on hemodialysis patients' self‐care deficits. Modern Care Journal, 9(4), 344–354.

[nop2412-bib-0037] Ottaviani, A. C. , Souza, É. N. , Drago, N. D. C. , Mendiondo, M. S. Z. D. , Pavarini, S. C. I. , & Orlandi, F. D. S. (2014). Hope and spirituality among patients with chronic kidney disease undergoing hemodialysis: A correlational study. Revista Latino‐Americana De Enfermagem, 22(2), 248–254. 10.1590/0104-1169.3323.2409 26107832 PMC4292610

[nop2412-bib-0038] Palutzian, R. F. , & Ellison, C. W. (1982). Loneliness, spiritual well being and the quality of life in loneliness: a sourcebook of current theory, research and therapy.

[nop2412-bib-0039] Pinto, S. M. O. (2012). A espiritualidade e a esperança da pessoa com doença oncológica. Estudo numa população de doentes em quimioterapia. 10–89.

[nop2412-bib-0040] Puchalski, C. , Ferrell, B. , Virani, R. , Otis‐Green, S. , Baird, P. , Bull, J. , … Sulmasy, D. (2011). La mejora de la calidad de los cuidados espirituales como una dimensión de los cuidados paliativos: El informe de la Conferencia de Consenso. Medicina Paliativa, 18(1), 20–40. 10.1016/S1134-248X(11)70006-4

[nop2412-bib-0041] Rambod, M. , Peyrovi, H. , Sareban, M. T. , & Mohebbi Nubandeghani, Z. (2008). Dietary and fluid adherence in hemodialysis patients. International Journal of Nanoscience, 23(67), 15–22.

[nop2412-bib-0042] Rezaie, M. , Seyyed Fatemi, N. , & Hosseini, F. (2008). Spiritual health in patients with cancer under chemotherapy. Quarterly Journal of Hayat, 14(3,4), 33–39.

[nop2412-bib-0043] Schiavon, C. C. , Marchetti, E. , Gurgel, L. G. , Busnello, F. M. , & Reppold, C. T. (2016). Optimism and hope in chronic disease: A systematic review. Frontiers in Psychology, 7, 2022 10.3389/fpsyg.2016.02022 28101071 PMC5209342

[nop2412-bib-0044] Shafipour, V. , Jafari, H. , Shafipour, L. , & Nasiri, E. (2010). Assessment of the relationship between quality of life and stress in the hemodialysis patients in 2008. Pakistan Journal of Biological Sciences: PJBS, 13(8), 375–379. 10.3923/pjbs.2010.375.379 20836297

[nop2412-bib-0045] Souliotis, K. , Alexopoulou, E. , Papageorgiou, M. , Politi, A. , Litsa, P. , & Contiades, X. (2016). Access to care for multiple sclerosis in times of economic crisis in Greece ‐ the HOPE II study. International Journal of Health Policy Management, 5(83–89). 10.15171/ijhpm.2015.173 PMC4737546 26927393

[nop2412-bib-0046] Taheri kharameh, Z. , Asayesh, H. , Zamanian, H. , Shoouri Bidgolisssss, A. , Mirgheisari, A. , & Sharififard, F. (2013). Spiritual Well‐being and religious coping strategies among hemodialysis patients. International Journal of Palliative Nursing, 1(1), 48–54.

[nop2412-bib-0047] Takaki, H. , & Yano, E. (2006). Possible gender difference in the relationships of self‐efficacy and the internal locus of control with compliance in hemodialysis patients. Behavioral Medicine, 32(1), 5–11. 16637257 10.3200/BMED.32.1.5-11

[nop2412-bib-0048] Theofilou, P. (2011). Anxiety and depression: A comparison between hemodialysis and kidney transplantation patients. Journal of Depression and Anxiety, 1, 1–2.

[nop2412-bib-0049] Theofilou, P. (2012a). Self‐esteem in Greek dialysis patients: The contribution of health locus of control. The Iranian Journal of Kidney Diseases, 6, 136–140. 22388613

[nop2412-bib-0050] Theofilou, P. (2012b). Sexual functioning in chronic kidney disease: The association with depression and anxiety. Hemodialysis International, 16, 76–81. 10.1111/j.1542-4758.2011.00585.x 22099520

[nop2412-bib-0051] Tutton, E. , Seers, K. , & Langstaff, D. (2012). Hope in orthopedic trauma: A qualitative study. International Journal of Nursing Studies, 49(7), 872–879. 22342114 10.1016/j.ijnurstu.2012.01.013

[nop2412-bib-0052] Unruh, M. , Evans, I. , Fink, N. , Powe, N. R. , & Meyer, K. B. (2005). Skipped treatments, markers of nutritional nonadherence and survival among incident hemodialysis patients. American Journal of Kidney Diseases, 46(6), 1107–1116. 10.1053/j.ajkd.2005.09.002 16310577

[nop2412-bib-0053] Yi, M. S. , Mrus, J. M. , Wade, T. J. , Ho, M. L. , Hornung, R. W. , Cotton, S. , … Tsevat, J. (2006). Religion, spirituality and depressive symptoms in patients with HIV/AIDS. Journal of General Internal Medicine, 21(5), 521–527. 10.1111/j.1525-1497.2006.00643.x 17083496 PMC1924785

